# Transcrestal Sinus Lift Procedure Approaching Atrophic Maxillary Ridge: A 60-Month Clinical and Radiological Follow-Up Evaluation

**DOI:** 10.1155/2015/261652

**Published:** 2015-09-16

**Authors:** G. Lo Giudice, G. Iannello, A. Terranova, R. Lo Giudice, G. Pantaleo, M. Cicciù

**Affiliations:** ^1^Medical Sciences and Stomatology Department, School of Dentistry, University of Messina, Via Consolare Valeria, 98100 Messina, Italy; ^2^Human Pathology Department, School of Dentistry, University of Messina, Via Consolare Valeria, 98100 Messina, Italy

## Abstract

*Aim. *The aim of this study was to assess the success and the survival rate of dental implants placed in augmented bone after sinus lifting procedures. *Material and Methods.* 31 patients were mainly enrolled for a residual upper jaw crest thickness of 3 mm. CBCT scans were performed before and after the augmentation technique and at the follow-up appointments, at 3, 6, 12, 24, and up to 60 months. The follow-up examination included cumulative survival rate of implants, peri-implant marginal bone loss, and the height of sinus floor augmentation. *Results.* This retrospective study on 31 patients and 45 implants later inserted in a less than 3 mm crest showed excellent survival rates (99.5%), one implant was lost before loading due to an acute infection after 24 days, and two implants did not osteointegrate and were removed after 3 months. The radiological evaluation showed an average bone loss of 0.25 mm (±0.78 mm) at the first follow-up appointment (3 months) up to 0.30 mm (±1.28 mm) after 60-month follow-up. *Conclusion.* In this study it was reported how even in less than 3 mm thick crest a transcrestal technique can predictably be used with a long-term clinical and radiological outcome, giving patients excellent stability of the grafted material and healthy clinical results.

## 1. Introduction

The jawbone resorption, related to the loose teeth, causes atrophy in the bone volume, by increasing the vertical dimension of occlusion and by reducing the amount of available bone to the implant placement and next prosthesis positioning. In presence of severe postextractive resorption, many techniques have been described to augment the residual bone ridge by using the possibility of the sinus membrane elevation up to 5 mm without any tearing [1]. The sinus lift technique was firstly described by Boyne and James [2] and it was based on a modification of the Caldwell-Luc sinus revision, basically consisting in a lateral approach to the sinus that allows a remarkable bone augmentation >10 mm even in very atrophic ridge [1]; this approach is well documented in literature and has proven to be safe and highly predictable.

In order to reduce morbidity and postoperative discomfort, Tatum Jr., in 1986, proposed a transcrestal approach for the sinus augmentation, using an osteotome sequence to have a controlled fracture of the sinus wall [3]. In 1994 Summers modified this technique, allowing a lateral force compression and increased the lateral bone density. This technique gave the clinicians the opportunity in having the implant site preparation using conical osteotomes [4, 5]. In 2000 Cosci and Luccioli proposed a 1 stage crestal approach using specific drill sequences (Cosci's Technique) and a particular tip able to prevent sinus perforation by using an abrasive removal of the cortical bone, without any fracture [6].

The transcrestal approach is considered the more conservative one and it has several advantages compared to the lateral osteotomy. Even though the transcrestal sinus lifting procedure is blindly performed, the frequency of sinus membrane perforation has been reported as less frequent than the lateral approach [7, 8].

The main goal of this procedure consists in a long time, no subjected to resorption, new bone formation, which allows a high predictable implant survival rate [9, 10].

This technique documented a 5-year survival rate superior to 92.7% for implants placed in less than 5 mm ridge height and 94.9% for implants inserted in more than 5 mm ridge height [11, 12]. These results are strictly linked to the absence of intraoperative and postoperative complication such as membrane perforation, postoperative sinusitis, disturbed wound healing, hematoma, sequestration of bone, and partial or complete graft failure [13, 14]. The absence of membrane perforation can be obtained by either performing a lateral sinus lift approach that allows directly checking the membrane status or, when a crestal approach is performed, gently detaching the membrane and checking, before the graft insertion, its integrity with the Valsalva manoeuvre [15]. Sinusitis might occur due to obstruction of the sinus outflow tract by mucosal edema and particulate graft and can be avoided thanks to a proper radiological evaluation and the absence of membrane perforation [16].

The aim of this retrospective study was to evaluate the survival rate of implants placed in the posterior upper jaw with a residual bone height less than 3 mm using a sequential sinus lift performed by crestal approach. By the five-year follow-up results of the investigations, it was also aimed at highlighting the effectiveness and the predictability of the performing sinus lift by occlusal window. The main limit of the sinus elevation surgery is the long-term follow-up control due to the grafted material resorption after several years. The stability of the grafted material and the clinical outcomes of the treated cases have been also recorded.

## 2. Materials and Methods

During the period from 2009 to 2014, 256 patients were referred to our Department for having dental implant rehabilitation in the posterior upper jaw region. About those procedures, a number of 64 patients needed bone augmentation by sinus lift surgery. 31 patients were enrolled in this retrospective study as described below.

All the patients, object of our study, 21 men and 10 women (mean age, 51.2), were partially edentulous and necessitate maxillary sinus elevation procedures for the implant placement. Additional inclusion criteria were as follows: residual bone height less than 3 mm, good state of health, absence of disease that affects wound healing or bone metabolism, and no regular medication consumption for >5 months. All the patients subjected to bone regeneration techniques were nonsmokers. Before implant placement, all patients received oral hygiene instruction and all of them were treated with nonsurgical periodontal therapy when considered necessary. All patients signed an informed consent form detailing the study procedures, according to the 2008 Helsinki protocols and the ethical requirements. These patients presented a bone height of 2 mm (±0.5), making sinus lift procedures necessary for the implant placement. The residual bone height was determined for each site by using a Cone Beam Computed Tomography (CBCT).

### 2.1. Radiographic Analysis

**Figure 1:**
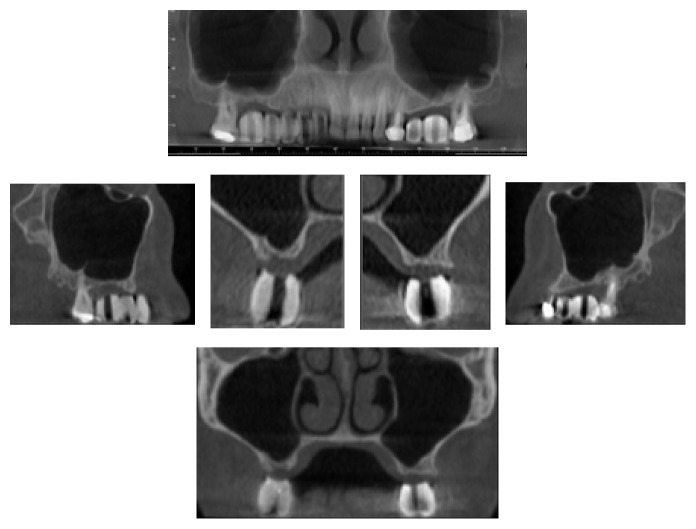
Baseline. The CBCT images show the posterior upper jawbone defect.

**Figure 2:**
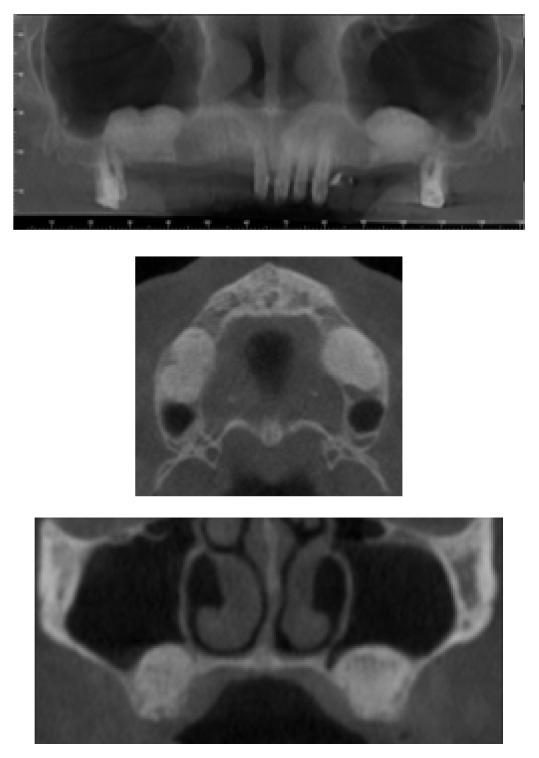
The sinus after the augmentation technique. It is possible to underline the material.

**Figure 3:**
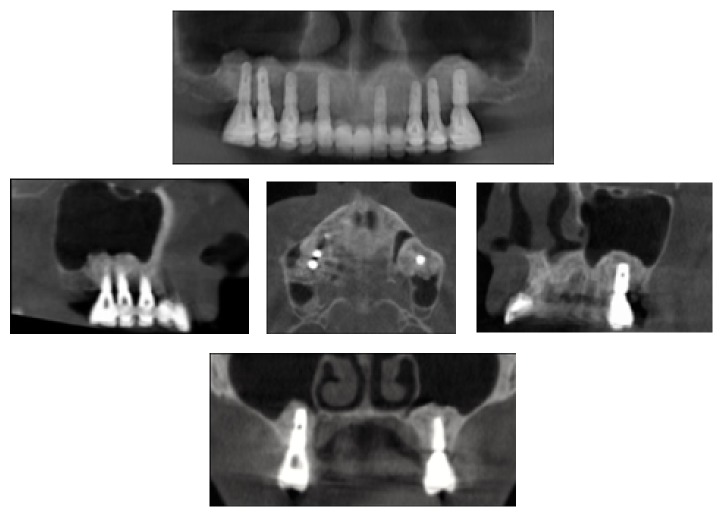
60-month follow-up of the augmented sinus.

Radiological images (SkyView CBCT Scanner from MyRay) were obtained before implant placement (baseline) (Figure 1), after the sinus augmentation technique (about 6 months post-op) (Figure 2) and the implant positioning (T1), after 3 months (T2), after 6 months (T3), after 12 months (T4), after 24 months (T5), after 48 months (T6), and 60 months (T7) (Figure 3).

The radiographic measurements were made after the 3 d digital reconstruction on the CTs axial section and sagittal or coronal reconstructions, considering the following parameters:

1. Residual bone height from the alveolar crest to the floor of the maxillary sinus (baseline).
2. Bone height at T1.
3. Bone height at follow-up appointments.

**Figure 4:**
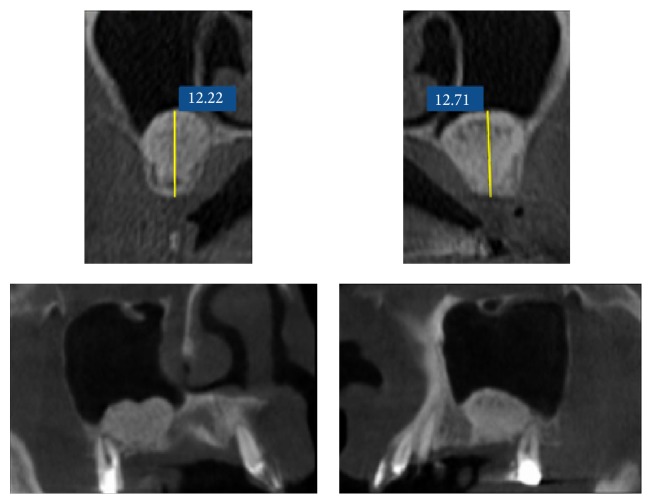
Radiological tridimensional evaluation of bone augmentation.

The radiological bone augmentation (BA) was calculated using the distance between the sinus floor and the occlusal alveolar ridge at the baseline and comparing it to the same distance at the time of examination (Figure 4).

(4) The height of the graft which was calculated measuring from the mesial and distal edges of the graft around the implant to the floor of the sinus.
(5) The graft reduction (GR) which was calculated making the difference between the height of the graft measured at the baseline and the height of the graft at the time of control X-ray analysis. The measure was performed at the same CBCT section for each patient.

Every measurement was calculated as mean value between mesial and distal measurement. A difference of <0.5 mm was considered as a clinically not significant discrepancy.

Peri-implant radiological bone loss, bone resorption, absence of bleeding, and possible signs of inflammation were evaluated during every follow-up appointment by clinical and CBCT evaluation accordingly with the radiological parameters being overstated. The patients included in this clinical study were treated by a single operator and gave their consent to perform the treatment as described in the paper.

### 2.2. Surgical Procedures

**Figure 5:**
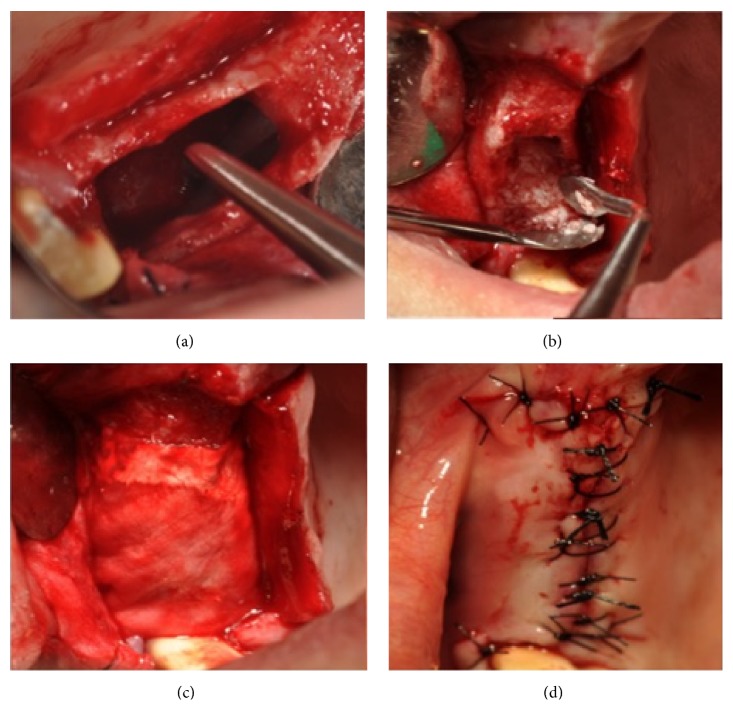
Clinical view of the sinus cavity (a). Cavity filled with autologous/heterologous material (b). Collagen sheet placed (c). Suture placed (d).

All patients were treated according to the following surgical protocol. All were premedicated with antimicrobial agent (amoxicillin and clavulanic acid 1 gr.) 1 hour before the surgery and continued the therapy for 5 days after, ibuprofen 100 mg twice daily for pain control, if needed, and 0.12% chlorhexidine digluconate mouthwash twice daily for 1 week for plaque control, starting one day after the surgery. A soft diet was recommended, avoiding contact of the surgically involved zone with food for a few days if possible. Patients rinsed their mouth with 0.20% chlorhexidine for 1 minute before surgery and under local anesthesia (mepivacaine 2%); a full-thickness flap was elevated; two vertical releasing incisions were made if necessary. According to the prosthetic treatment planning, the location for implant placement was established. Bone incision was made with a piezoelectric device (Mectron Piezosurgery) using one cutting bur and dislocating the bone fragment along with the detached Schneiderian membrane into the new upper position. The lifting movement of the membrane without trauma was ensured by using a noncutting divaricator. To ensure the absence of perforation, the Valsalva manoeuvre was performed, confirming the integrity of the membrane, compared to the graft, a mixture of the autologous bone and Geistlich Bio-Oss (Geistlich Biomaterials Italia S.r.l., VI) was gently pushed elevating the already detached membrane, and this step was repeated until the whole site was filled as planned. An intraoral X-ray was made to check the height of the graft. The site was covered with a slow resorbable membrane Geistlich Bio-Gide (Geistlich Biomaterials Italia S.r.l., VI) and sutured with a SUPRAMID NYLON 4/0 nonabsorbable suture (Lorca Marine ES) (Figure 5).

Sutures were removed one week after surgery.

The implants OSSTEM TSIII SA (OSSTEM, KO) were inserted in the first molar position, in the second molar position, and in the second premolar position (Table 1); positioning was made after 6 months, leaving all the implants submerged. After six months the implants were exposed with transmucosal healing abutments and functionally loaded after 2 weeks. All implants had to be in function for a minimum of 48 months.

### 2.3. Statistical Analysis

The quantitative data (baseline-T7) were expressed as average ± SD. The Student *t*-test (for paired samples) was used to evaluate the statistical difference. Statistical significance was set at *P* < 0.05.

**Figure 6:**
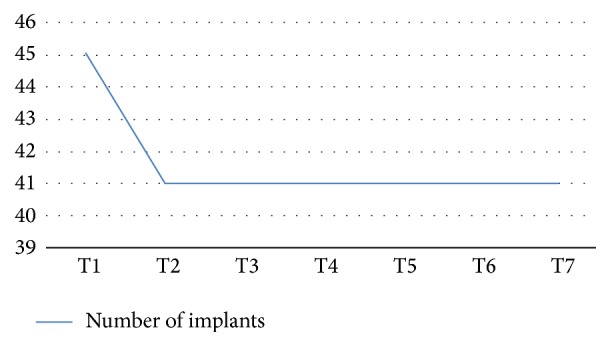
Implant survival rate.

Implant survival was expressed as the percentage of lost implants in relation to the total number of implants inserted. The data were analysed using Kaplan-Meier analysis to provide cumulative survival rates [17, 18] (Figure 6).

## 3. Results

The first check to the patients was performed at 6 months after the sinus lift surgery performing a new CBCT. The CBCT baselines of each patient and the radiological stents guide have been used for measuring the grafted material presence after the surgery. Moreover, the patient underwent the CBCT by using the same radiologic machine in order to have less bias at the time of the evaluation. During the 5-year period (2009–2014), 45 implants were inserted in 31 patients. The average follow-up time was 52 months (±12 SD; range 0–60 months).

**Figure 7:**
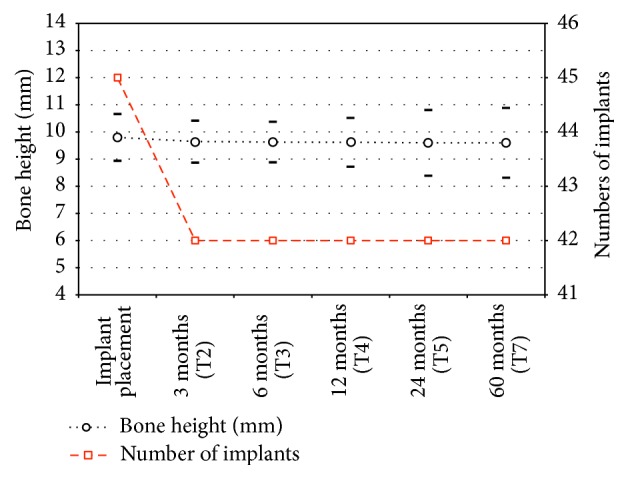


The implants placed were inserted in the first molar position, in the second molar position, and in the second premolar position accordingly with the values recorded on the Table 1. All the implants used were 4 mm in diameter and number 11 was 8.5 mm in length and number 34 was 10 mm in length (Table 2). The cumulative survival rate of the implants was 99.5%. Of the 45 implants placed, a total of 3 were lost: 1 was lost before loading due to an acute infection after 24 days. Two implants did not osteointegrate and were removed after 3 months (Figure 6). No other adverse effects were observed. The average bone height, considered from the alveolar crest to the bottom of the implant, at the time of implant positioning (T1) was 9.8 mm (±0.86 mm). The measured average bone height at the first follow-up appointment (T2) was 9.65 mm (±0.78 mm), with an average bone loss of 0.25 mm. The marginal bone loss of each implant was measured both mesially and distally; the range of loss was from 0 to 2 mm, showing and average value of 0.2 mm (±0.2 mm) mesially and 0.3 mm (±0.15 mm) distally (Figure 7 and Table 3).

## 4. Discussion

The sinus augmentation procedure has been demonstrated as being a reliable and sometimes a mandatory technique when rehabilitating a maxillary atrophic ridge with pneumatized sinuses. The lateral approach proposed by Boyne and James in 1980 allowed a remarkable bone increasing >10 mm, even in atrophic ridges, however resulting in a significant higher postsurgical morbidity and an increased risk of membrane perforation [1, 2, 7]. Crestal approach, osteotome mediated sinus lift surgery, may be performed with different bone grafting material, such as allograft, autogenous bone or heterologous materials, and platelet derivatives themselves or combined with grafting materials, in order to combine the properties of the growth factor to the mechanical presence of soft platelet derivate that allows a better force control during the sinus floor elevation [19–22].

The two augmentation techniques are designed for different clinical situation; Rosen et al. showed how the survival rates for the Summer's technique are strictly linked to the residual bone height, starting from 96% when 5 mm or more of bone is present, dropping to 85% when 4 mm or less is present; however these results may be more linked to the primary stability of the implant than to more biological reasons [23, 24].

The bone height has a relevant influence on the survival rates of implant positioned on augmented bone, decreasing its value with reduced bone height [24, 25].

A decreased bone height resorption rate might be influenced by the osteotomy technique that can maintain a better cellular vitality especially when piezoelectric devices are used instead of rotating instruments [26, 27].

In 1986, Tatum Jr. proposed a transcrestal, more conservative, approach later modified by Summers that first described the use of osteotomes to elevate the membrane and eliminate hammering, making the technique more comfortable for the patient. The crestal technique is nowadays a reliable method allowing contextual implant insertion with good survival rates. However the necessary height of >5 mm of residual bone height due to the risk of membrane perforation and a low implant stability were the main limitation of this technique. In the present retrospective study, the 45 implants later inserted in a 2 mm crest showed excellent survival rates (99.5%), calculated in a significant follow-up period (60 months). This higher result, if compared to other retrospective reports, can be explained thanks to a reduced risk of membrane perforation thanks to the use of piezoelectric device [26]. This outcome is evident considering the bone height gain (7.8 mm, ±0.86 mm) which is greater than the average of the osteotome technique. This outcome may also be attributed to the nonsmoker selection of patients due to the evidence of the negative impact on bone healing of the nicotine [28].

A reduction of the grafted material has been reported over the first three months of bone remodeling and remained stable over the whole follow-up period (60 months). Other reports showed lower 5-year survival rates of the dental implants placed (97.83, 95.45). However, they consider a higher number of implants [29, 30].

The results observed could be favourably compared with the observation from similar study in which implants were placed into a severely resorbed ridge, with less than 4 mm of residual bone height [31].

Other studies reported lower implant survival rates from 96% to 85.7% when the residual bone height was 4 mm or less, considering the height of bone from the crest of the alveolar ridge to the sinus floor as the most important factor affecting the implant survival rate; this concept is strictly linked to the necessity to insure a high primary stability especially in severe atrophied ridge [12, 23].

More recent studies from Gonzales underlined the good long-term predictability of this technique even in case of simultaneous implant placement in patients with residual bone height of 4 mm or less, confirming that the residual bone height did not increase crestal bone loss or reduce the success rate of the implants and associated prostheses [32].

In particular Mazor et al. showed a 100% implant survival rate at 18-month follow-up, demonstrating the safety and predictability of this minimally invasive sinus lift elevation technique [33].

One of the main aspects that must be considered for a long-term success, especially observed in this study, is linked to the ability to elevate the Schneiderian membrane without any tearing, in addition to a correct anatomy evaluation, a low membrane detachment force, and elasticity and deformation capacity judgment. An increased number of insertion sites can increase the membrane elevation height increasing the elastic properties of the Schneiderian membrane.

The use of grafting materials is, however, debated with several authors describing a consistent bone formation (6.51 mm ± 2.49 mm) even when no grafting material was used after a minimum of 1-year follow-up [34] and others suggest their necessity as the use of a blood clot or platelet concentrates alone may lead to unpredictable results [35]. When grafting materials were used the autologous bone representing nowadays the gold standard however might be subjected to extensive resorption and might be linked to endosinusal contamination due to intraoral pathogens [36, 37].

The height of bone gain is comparable to the one achieved with lateral approach while maintaining the advantage of a less invasive approach with less postoperative morbidity [38].

Our data confirm that the crestal augmentation technique gives the surgeon the possibility of a big bone height augmentation with good long-term survival rates, allowing the insertion of adequate implants per length and diameter, as suggested in literature, even in extreme atrophic ridge.

Further clinical and* in vitro* investigations are needed to measure the mechanical properties of the Schneiderian membrane, minimum force needed for its detachment from the underlying bone and its elasticity and load limits.

## 5. Conclusion

This analysis suggests that the crestal approach is a successful bone augmentation technique even in a severe atrophic maxilla with 2 mm of crestal bone height.

## Figures and Tables

**Table 1:** Implant positions (*n* = 45).

Teeth	1.5	1.6	1.7	2.5	2.6	2.7

Implant	3	20	6	5	7	4

**Table 2:** Implant dimension (*n* = 45).

Diameter (mm)	Length (mm)	
	8.5	10

4	11	34

**Table 3:** Mean vertical bone heights (mean follow-up 52 ± 12; range, 0–60 months; *n* = 45 = implants). See Figure 7.

Bone height	Implant placement	3 months (T2)	6 months (T3)	12 months (T4)	24 months (T5)	60 months (T7)
	9.8 mm (±0.86 mm)	9.65 mm (±0.78 mm)	9.63 (±0.75 mm)	9.62 (±0.90)	9.60 (±1.21)	9.60 (±1.28)

*n*	45	42	42	42	42	42
